# Divergent Functional Roles of Shredder Size: Interspecific Versus Intraspecific Effects on Aquatic Leaf Litter Decomposition

**DOI:** 10.1002/ece3.72907

**Published:** 2026-01-12

**Authors:** Mourine J. Yegon, Pratiksha Acharya, Katrin Attermeyer, Wolfram Graf, Simon Vitecek

**Affiliations:** ^1^ Wassercluster Lunz—Biological Station Lunz am See Austria; ^2^ Institute of Hydrobiology and Aquatic Ecosystem Management, University of Natural Resources and Life Sciences Vienna Austria; ^3^ Department of Functional and Evolutionary Ecology University of Vienna Vienna Austria; ^4^ Department of Ecology University of Innsbruck Innsbruck Austria

**Keywords:** FPOM particle sizes, ontogenetic stages, particle size distribution, shredder diversity, shredder identity

## Abstract

Biodiversity underpins ecosystem functioning, and higher diversity of taxa and traits often enhances efficiency. However, such relationships may vary, as intraspecific variation—including differences across ontogenetic stages—can modulate a taxon's contribution to ecosystem functioning. We conducted laboratory experiments to examine the effects of shredder identity, diversity, and ontogenetic stage on leaf litter decomposition and fine particulate organic matter (FPOM) production. Three caddisfly shredder taxa (*Allogamus*, *Potamophylax*, and *Sericostoma*) were collected in two different months and fed with highly decomposable alder ( *Alnus glutinosa* ) leaves individually and in shredder combinations during 1‐week incubation periods. The experiments reflected distinct larval instars and were conducted with a time interval of 2 months. We hypothesized that decomposition and FPOM production would vary with taxon identity and ontogeny, with diversity enhancing processing rates. We further expected higher processing rates in younger larvae due to their greater metabolic demands. Results showed that both shredder identity and ontogenetic stage significantly affected decomposition, FPOM production, and particle size distribution. The largest taxon, *Potamophylax*, had the highest decomposition and FPOM production rates and produced the largest FPOM particles, while the smallest taxon, *Allogamus*, had the lowest rates and produced the smallest particles. Within taxa, younger and smaller larval stages exhibited higher rates than their older conspecifics. These results highlight the importance of shredder identity and ontogenetic stage in shaping both the magnitude and timing of key ecosystem processes.

## Graphical abstract

Biodiversity enhances stream ecosystem efficiency, with greater taxonomic and trait diversity improving functions like decomposition. Shredder diversity arises from both species differences and ontogenetic variation. We experimentally examined these factors to reveal how they shape the magnitude and timing of organic matter processing in streams.

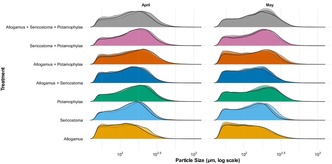

## 1 Introduction

In stream ecosystems, biodiversity is a critical driver of ecosystem functioning, and greater diversity is often linked to greater efficiency in processes like nutrient cycling and organic matter breakdown (Palmer 1997; McKie et al. 2009; Swan and Kominoski 2012). Macroinvertebrate shredders in particular play an important role in the decomposition of particulate organic matter, thereby accelerating nutrient cycling and energy flow through food webs (Wallace and Webster 1996; Gessner et al. 2010). However, while the general role of shredders in leaf litter breakdown is well established, the individual contributions of single taxa to decomposition and fine particulate organic matter (FPOM) production and particle size distribution are less well understood (Bradford et al. 2002; Santonja et al. 2018). Understanding the role of individual taxa is important because some taxa consume and process detritus more efficiently than others, suggesting the existence of varying functional traits among shredders (Boyero et al. 2007; Dangles et al. 2011; Balik et al. 2022).

These functional differences arise from interspecific variation among species and intraspecific variation within species, reflected in traits such as body size, metabolic demand, and feeding physiology (Tonin et al. 2018; Balik et al. 2022). Although closely related organisms are often presumed to be functional equivalents, this is not always the case. Variation in these functional traits, including differences in life history strategies and developmental phenology, can lead to substantial differences in detritus processing efficiency (Wissinger et al. 2003; McKie et al. 2008; Merritt et al. 2017). For example, Nolen and Pearson (1993) showed that younger instars of *Anisocentropus kirramus* were unable to feed on tougher leaves, yet processed softer material at a higher rate per unit body mass than older instars. These traits not only regulate the rate of leaf litter breakdown but may also influence the production of FPOM, a key energy source for collectors in aquatic ecosystems (Vannote et al. 1980; Callisto and Graça 2013; Bundschuh and McKie 2016).

The breakdown of leaf litter by shredders to generate FPOM has been shown to vary (Friberg and Jacobsen 1994; Dangles and Malmqvist 2004; Halvorson et al. 2015), with FPOM production being reported to vary up to 20‐fold across macroinvertebrate species in terms of the mass produced per unit time, and directly related to their rates of litter consumption (Santonja et al. 2018). Understanding these species‐specific contributions is essential, particularly in the face of biodiversity loss, which may alter assemblage composition and disrupt key ecosystem functions (Johnson 2000; Gessner et al. 2010; Wardle et al. 2011; Pacini et al. 2024). Beyond species identity that often reflects differences in functional traits such as body size (Rudolf et al. 2014), ontogenetic shifts play a crucial role in shaping decomposition dynamics. Younger instars typically exhibit higher metabolic rates (Friberg and Jacobsen 1999), driving faster leaf breakdown and higher FPOM production. Also, smaller organisms with shorter lifespans often grow proportionally faster than larger ones (Peters 1986; Balik et al. 2022).

Based on the metabolic‐scaling theory (Brown et al. 2004), smaller taxa should process leaves faster (per unit biomass) than larger taxa because they have faster metabolism. However, the advantages of greater size, strength, or morphological adaptations in the mouthparts might outweigh the effect of metabolism on leaf processing rates, making predictions based on metabolic scaling inappropriate when comparing phylogenetically different groups (Patrick 2013). As shredders mature and grow, shifts in feeding behavior and physiology may also alter their capacity to process organic matter (e.g., Friberg and Jacobsen 1994; Céréghino 2006). While younger individuals are often more metabolically active and process leaves faster due to higher metabolic rates, older instars become more specialized in their traits. For example, as insects grow, their mandibles and other mouthparts typically become more developed and efficient, enhancing their ability to break down tougher or more recalcitrant leaf litter, which may be particularly important when leaf litter inputs are low (Friberg and Jacobsen 1994; Céréghino 2006). This dual influence of metabolic rate and morphological specialization raises an important question: how do ontogenetic shifts in addition to differences in body size influence leaf litter processing in streams? Obviously, this question implies that the role of shredders in organic matter processing may not be uniform across species or life stages (Tank et al. 2010). Consequently, the timing and intensity of ecosystem functioning may vary depending on which taxa are active at different developmental stages.

Shredder diversity can further influence decomposition dynamics through complementarity and facilitation effects, where species with different functional traits enhance resource use efficiency (Creed et al. 2009). Well‐established diversity‐function studies predict that positive diversity effects, driven by niche or resource partitioning and facilitation, can counteract the negative interactions between species (Hooper et al. 2005; Vos et al. 2011). However, diversity effects are not always positive; competition and selection pressures may reduce decomposition efficiency in some species combinations (Creed et al. 2009; Gessner et al. 2010). Given these complexities, it is crucial to explore how species identity, developmental stage, and biodiversity interact to regulate decomposition processes in streams.

Thus, in this study, we examined how macroinvertebrate shredder identity, ontogenetic stage, and diversity influence leaf litter decomposition and FPOM production. To facilitate this, we used three case‐building caddisfly larvae at different ontogenetic stages: two from one family (Limnephilidae; *Potamophylax* and *Allogamus*) and a third from a separate family (Sericostomatidae; *Sericostoma*) to extend our functional diversity. The three taxa differ in several traits, including body size and mandible morphology. *Potamophylax* larvae are relatively large and have broad, toothed mandibles adapted for scraping periphyton off leaf surfaces and shredding coarse leaf material (Otto 1971; Waringer et al. 2013). *Allogamus* larvae are smaller than *Potamophylax* and possess similarly toothed mandibles for shredding and scraping organic material, though subtle differences in mandible shape may affect how they process leaf litter (Waringer et al. 2013). *Sericostoma* larvae are medium‐sized, construct smoother, curved cases, burrow in the substrate, and have mandibles capable of both scraping periphyton off leaf surfaces and biting through leaf material (Crichton 1957; Iversen 1973).

We expected interspecific differences in feeding behavior, morphology, and nutritional requirements to drive variation in decomposition rates and FPOM production. Accordingly, we tested three main hypotheses: (1) Shredder identity—reflecting a body size gradient—significantly affects decomposition rates, FPOM production, and FPOM particle size, with larger individuals expected to decompose leaves faster and produce FPOM at higher rates, consequently generating smaller FPOM particles due to more efficient processing; (2) larval instar—reflecting ontogenetic changes in body size—significantly influences decomposition rates, FPOM production, and FPOM particle size, with smaller, earlier instars exhibiting higher decomposition and FPOM production rates, and generating smaller FPOM particles due to elevated metabolic activity; (3) increased shredder diversity—corresponding to a greater functional trait space—enhances leaf processing efficiency, as shown by higher decomposition rates, greater FPOM production, and a more diverse FPOM particle size distribution.

We tested these hypotheses using a factorial experimental design that manipulated shredder identity, ontogenetic stage, and diversity, feeding individuals from three shredder taxa with highly decomposable alder leaves ( *Alnus glutinosa* , hereafter alder).

## 2 Materials and Methods

### 2.1 Experimental Design and Setup

This study was conducted in a climate‐controlled chamber at WasserCluster biological station, Austria. Our experimental setup consisted of a completely randomized factorial design incorporating a first factor “shredder taxa” with seven levels created by the three shredder taxa (*Allogamus*, *Sericostoma*, and *Potamophylax*), their pairwise mixtures (*Allogamus + Sericostoma, Allogamus + Potamophylax*, *Sericostoma* + *Potamophylax*), and their three‐shredder combination (*Allogamus + Sericostoma* + *Potamophylax*) crossed with a second factor “sampling month” with two levels (April and May, 2025). The resulting total of seven treatments in each month was replicated five times in 35 independent bench‐top microcosms (food‐grade plastic buckets, 1100 mL volume, bottom diameter 122 mm) filled with 700 mL stream water from Oberer Seebach, Austria (47°51′ N, 15°04′ E, 609 m a.s.l.). The design and replication structure of the experiment are summarized in Table S1.

The water used in the microcosms was collected 1 day prior to the experiment and then filtered through a 500 μm mesh hand filter. During the experiment, the water in all microcosms was aerated and maintained at 12°C in a climate chamber under an 11:13 h dark: light photoperiod. We installed a 1 mm steel mesh halfway up each microcosm to suspend the shredders and leaf material. This setup ensured that only FPOM < 1 mm, produced during leaf processing, passed through the mesh and accumulated at the bottom of the microcosm (Figure S1). Each experiment lasted 7 days, from shredder collection in the stream to final sampling. The 5‐day feeding phase was conducted in early April and late May to target different larval instars. At the end of each experiment, remaining leaf fragments (> 1 mm) were collected and stored at −70°C for subsequent leaf mass loss and decomposition rate analyses.

### 2.2 Leaf Resources

Prior to the start of the experiment, alder leaves were collected as freshly abscised material in Lunz am See, Austria, where the experiment was conducted, air‐dried, and stored at room temperature in dry, dark boxes until use. Damaged, decayed, or diseased leaves were removed to ensure consistency of the experimental material. We fed the shredders oxically conditioned alder leaf discs, 12 mm in diameter (approximately 1.1 cm^2^ surface area), cut from leaves that were lightly moistened using a cork borer; subsequently, the discs were air‐dried at room temperature for approximately 2 days prior to weighing. For each treatment, we used leaf discs with a dry mass of 1.0 ± 0.003 g. To condition the leaf discs as food for the shredders, we allowed them to be colonized by microbes under oxic conditions by placing them in aerated stream water from the same source as used in the main experiment, in the dark at 12°C for 7 days. To ensure consistency in microbial communities, the leaf discs were shaken every 3 days. Surplus leaf discs were prepared and used for leaf mass loss corrections because of handling and leaching.

### 2.3 Invertebrate Shredders

The shredder larvae used in the study—*Allogamus*, *Sericostoma*, and *Potamophylax—were* collected from Oberer Seebach in Lunz am See (47°51′ N, 15°04′ E) and transported to the experimental units in the climate chamber. These three taxa are all caddisflies (Trichoptera) and share the shredder feeding role, but belong to different families: *Sericostoma* (Sericostomatidae) and *Allogamus* and *Potamophylax* (Limnephilidae). All three taxa have a univoltine life cycle and are abundant in the pre‐alpine headwater streams near the research station.

In the climate chamber, the larvae were immediately placed in filtered (GF/F, 0.7 μm pore size) stream water in plastic cups and allowed to defecate for 24 h to empty their guts before the start of the experiment and prior to the biomass measurements in early April and late May. To track developmental changes, we used 30 additional specimens that were collected, freeze‐dried (Genesis freeze dryer, Virtis Inc., NY, USA), and measured to assess initial shredder biomass (caseless organisms' biomass, case removed after freeze‐drying) and head capsule width (HCW). These control biomass and HCW measurements were obtained at the start of each experiment in early April and late May (Figure S2). We used HCW and biomass as proxies for the key ecological trait of body size, reflecting differences across ontogenetic stages. HCW is a widely adopted measure in insect ecology due to its strong correlation with total body mass and its reliability in capturing size differences across developmental stages (Chown and Gaston 2010), while offering the advantage of being easily and accurately measurable (Benke et al. 1999). While some shredder taxa showed overlapping instar stages between April and May, increases in HCW and biomass still reflected meaningful developmental progression likely to influence feeding behavior and leaf processing. In *Sericostoma*, we observed slightly greater variability in older (May) instars, likely due to the onset of pupation during this period. This limited our ability to consistently sample the largest individuals, resulting in the inclusion of some smaller‐than‐expected larvae for that time point.

For the single shredder larvae treatments, 12 larvae were placed in each microcosm. In the shredder combinations, six (for the two taxa combinations) or four (for the three taxa combinations) of the larvae of each taxa were used to have a total of 12 specimens in each microcosm (Figure S1). We monitored and recorded the survival rates throughout the experimental period. Overall, shredder loss was minimal, with < 2% mortality observed over the course of the experiment, and mortality was not disproportionately associated with any taxa or treatment.

At the end of the experiment, we collected the surviving larvae and leftover leaf fragments and stored them at −70°C. We freeze‐dried all leaf and larvae samples from both the start and end of the experiment and weighed them to the nearest 0.001 mg using a microbalance (Sartorius CPA2P, Sartorius Lab Instruments GmbH & Co. KG, Göttingen, Germany).

### 2.4 FPOM and Leaf Sampling and Processing

At the end of the experiment, we carefully removed the steel mesh screen and allowed the particles to settle for 1 h (observed as sufficient from observations during pre‐tests). Afterward, about 70% of the water was removed from each microcosm using a syringe to minimize disturbance of the settled particles. The FPOM was then carefully pipetted from the bottom of the microcosm into sterile 15 mL centrifuge tubes (STARLAB Intl, Hamburg, Germany). To ensure that all FPOM was collected, the microcosm was carefully rinsed with MilliQ water, which was then added to the collecting tubes. Collected FPOM samples were stored at −70°C pending analyses of FPOM production rates, conversion efficiency, and particle size. Prior to processing, samples were thawed, their volumes recorded, and adjusted to a uniform volume by adding MilliQ water. Each sample was then gently homogenized by repeated tube inversions. To measure particle size distribution, a 5 mL subsample was subsequently pipetted into a petri dish for particle imaging using a KEYENCE microscope (Keyence VHX‐5000; Keyence GmbH, Neu‐Isenburg, Germany). We used the program Fiji (Schindelin et al. 2012) to measure the area, perimeter, and Feret's diameter of particles covering a known fraction of the petri dish using scaled images. Images were converted to 8‐bit grayscale, and particles were detected by applying a lower threshold of 5 and an upper threshold of 90 pixels, where 1 pixel was equivalent to approximately 10 μm. After this analysis, we refroze the subsamples for later freeze‐drying to measure FPOM dry mass on a micro‐balance to the nearest 0.001 mg. We calculated FPOM production rates for each treatment and each of five replicates over the entire experimental period based on the mass of generated FPOM per shredder and per unit time. Particle size distributions of the FPOM were measured for each replicated microcosm.

Relative leaf mass loss (LML) was calculated as the difference between the initial leaf mass (before leaf conditioning) and the final leaf mass at the end of the experiment, expressed as a percentage of the initial mass. We then calculated leaf decomposition rates from the LML using Equation (1):

(1) $$k=\frac{\ln\left(M_{0}\right)-\ln\left(M_{t}\right)}{t\times D}$$

where M_0_ represents the initial leaf dry mass, and M_*t*_ is the dry mass at time *t* (days of the experiment) and *D* is the shredder density (individuals per microcosm). This yields a rate constant expressed as per shredder per day. We also expressed FPOM production as a percentage of LML to quantify how much lost leaf mass is converted into FPOM (Equation 2) and call this variable FPOM conversion efficiency (FCE).

(2) $$\text{FPOM conversion efficiency}\;\left(\mathrm{FCE}\right)\;\left(\%\right)=\frac{\;\text{FPOM produced}\;\left(g\right)}{\text{Leaf mass lost}\;\left(g\right)}*100$$

### 2.5 Data Analysis

We tested for differences in response variables—decomposition rates, FPOM production rates, FCE, and mean of FPOM particle sizes—among shredder treatments (i.e., different shredder taxa and their combinations; seven levels), between the months of April and May, and for the interaction between shredder treatment and instar month. Analyses were conducted using Generalized Linear Models (GLM; function *glm*, *stats* package; R Core Team 2023) with a Gaussian error distribution and identity link function. GLMs included an interaction term between shredder treatments and instar month and were followed by post hoc pairwise comparisons using Tukey's Honest Significant Difference (HSD). Bartlett's test was used to check homogeneity of variances across treatments. To meet the assumptions of normality, decomposition rates, FPOM production rates, and mean FPOM particle sizes were log‐ or square‐root‐transformed prior to analysis. All data are reported as mean ± SD unless otherwise indicated. To describe the distribution of FPOM particle sizes, we generated ridgeline plots based on Feret's diameter for each treatment. We used R (version 4.2.3; R Development Core Team, Vienna, Austria, see http://www.r‐project.org/) for all statistical analyses.

Net diversity effects (NDE) were computed to assess how the shredder combinations influence the decomposition rates. This was obtained by computing the difference between the observed decomposition of shredder combination treatments (Obs.) and its expected value (Exp.), which is derived from the mean of the single shredder treatments (Rubio‐Ríos et al. 2021). Here, net diversity effects indicate whether leaf litter consumption is higher or lower in mixtures of shredder taxa than in single‐taxa treatments. We then applied a similar observed‐versus‐expected comparison to FPOM production rates and particle sizes. To account for potential differences in body size among the shredders, decomposition and FPOM production rates were analyzed in relation to shredder biomass. Biomass‐weighted decomposition and FPOM production rates were calculated by normalizing shredder‐specific decomposition rates based on their relative biomass. This approach corrects for differences in shredder size and ensures that decomposition contributions are assessed in proportion to shredder biomass. This adjustment ensured that any observed differences in net diversity effects could be attributed to factors other than size‐related differences in decomposition potential. To assess statistical differences among treatments, we applied GLMs to the net diversity effect values for decomposition, FPOM production, and mean FPOM particle size (function glm, *stats* package; R Core Team 2023). To test whether increased shredder diversity, corresponding to a greater functional trait space, enhanced leaf processing efficiency, FPOM production rates, and FPOM particle sizes, we calculated the functional richness metric (FRic) using the FD::dbFD package in R (Laliberté and Legendre 2010), which reflects the volume of functional trait space occupied by the taxa in each treatment. FRic was calculated from two continuous shredder traits: biomass and HCW, measured for each taxon. We then linked FRic values to ecosystem process variables using linear mixed‐effects models, with shredder identity included as a random factor, to assess whether FRic or shredder identity better explained variation in ecosystem processes.

Finally, we fitted linear regression models using *the lm()* function in the *stats* package (R Core Team 2023) to explore the relationships between shredder size (HCW) and both decomposition and FPOM production rates. Adjusted *R* ^2^ values were used to compare the predictive power of the models, and significance levels were corrected for multiple comparisons using a Bonferroni adjustment.

## 3 Results

Among the three taxa, *Potamophylax* exhibited the largest body size overall (Figure S2). In April, larvae were predominantly in early instar stages based on our HCW measurements compared with taxon‐specific published instar criteria. *Sericostoma* larvae were classified as early instar V (HCW = 1.96 ± 0.04 mm) following Iversen (1973), *Potamophylax* as instar IV (HCW = 2.18 ± 0.03 mm) following Otto (1971) and *Allogamus* as early instar V (HCW = 1.57 ± 0.05 mm) following Waringer (1989).

By late May, larvae had progressed to later instars: *Sericostoma* late instar V (HCW = 2.03 ± 0.05 mm; Wagner 1990; Iversen 1973), *Potamophylax* instar V (HCW = 2.23 ± 0.03 mm; Otto 1971) and *Allogamus* late instar V (HCW = 1.68 ± 0.02 mm; Waringer 1989). Biomass measurements showed corresponding increases, supporting the observed developmental progression (Figure S2). However, HCW and biomass did not consistently increase between consecutive instars, reflecting variability in growth within instars (Figure S2).

**FIGURE 1:**
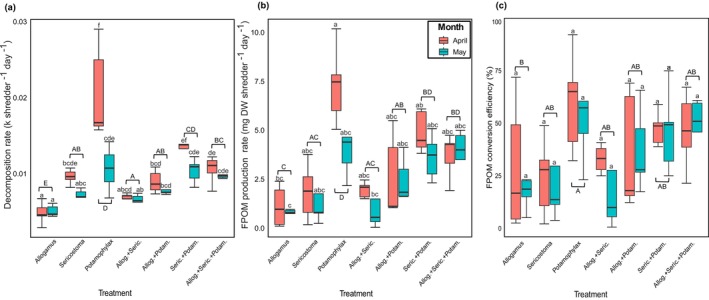
Decomposition rates (a), FPOM production rates (b), and FPOM conversion efficiency (c) across different treatments in the younger (April) and older (May) instar stages. Boxplot color denotes the instar sampling month. Different lowercase letters indicate significant differences among all “shredder treatment: month” interactions, and uppercase letters denote differences among shredder treatments. Allog. + Seric. = *Allogamus* + *Sericostoma*, Allog. + Potam. = *Allogamus* + *Potamophylax*, Seric. + Potam. = *Sericostoma* + *Potamophylax*, Allog. + Seric. + Potam. = *Allogamus* + *Sericostoma* + *Potamophylax*, FPOM = Fine Particulate Organic Matter.

Decomposition and FPOM production rates differed among shredder treatments (*F*_(6,56)_ = 34.26, *p* < 0.05; *F*_(6,56)_ = 20.09, *p* < 0.05, respectively; Figure 1, Table 1). Differences were also observed across shredder instars between months for both decomposition rates (*F*_(1,56)_ = 15.13, *p* < 0.05; Table 1) and FPOM production rates (*F*_(1,56)_ = 4.77, *p* < 0.05; Table 1). For decomposition rates, the interaction between shredder treatments and instar month was significant (*F*_(6,56)_ = 3.99, *p* < 0.05; Table 1).

**TABLE 1:** Results from GLMs to assess effects of shredder treatments, instar month, and their interaction on decomposition rates, FPOM production rates, and FPOM particle sizes.

Variables	Factors	Df	SS	Mean SS	F‐statistic	*p*
Decomposition rates	Shredder treatment	6	7.99	1.33	34.26	**< 0.001**
	Month	1	0.78	0.78	20.09	**< 0.001**
	Shredder treatment: Month	6	0.93	0.15	3.99	**0.002**
	Residuals	56	2.18	0.04		
FPOM production rates	Shredder treatment	6	17.67	2.94	15.13	**< 0.001**
	Month	1	0.93	0.92	4.77	**0.033**
	Shredder treatment: Month	6	0.94	0.16	0.81	0.567
	Residuals	56	10.9	0.19		
FPOM conversion efficiency	Shredder treatment	6	9526	1587.6	3.081	**0.0112**
	Month	1	102	102.2	0.198	0.6578
	Shredder treatment: Month	6	577	96.2	0.187	0.9794
	Residuals	56	28,860	515.4		
Mean FPOM particle size	Shredder treatment	6	16,410	2734.9	36.65	**< 0.001**
	Month	1	434	434.4	5.82	**0.019**
	Shredder treatment: Month	6	867	144.5	1.94	0.091
	Residuals	56	4179	74.6		

*Note:* Statistically significant (*p* < 0.05) values are shown in bold.

Abbreviations: df = degrees of freedom, MS = mean of squares, SS = sum of squares.

Decomposition rates varied depending on the shredder treatment × month interaction (GLM, *p* < 0.05). Intraspecific differences in decomposition rates were observed between younger (April) and older (May) instars. For *Potamophylax*, younger instars decomposed leaf material significantly faster than older instars (0.021 ± 0.003 vs. 0.011 ± 0.001 k/shredder/day). A similar pattern was found for *Sericostoma* (0.014 ± 0.001 vs. 0.010 ± 0.001 k/shredder/day) and for the *Sericostoma* + *Potamophylax* combination (0.016 ± 0.001 vs. 0.013 ± 0.001 k/shredder/day) (Tukey‐adjusted comparisons, *p* < 0.05; Table S2). In contrast, no significant intraspecific differences between April and May instars were observed for *Allogamus*, the *Allogamus* + *Potamophylax* and *Allogamus* + *Sericostoma* combinations, or the three‐taxa combination (*Allogamus* + *Sericostoma* + *Potamophylax*) (Table S2). Across all taxa, interspecific differences in decomposition rates were observed, with younger (April) instars of *Potamophylax* exhibiting the highest rate overall, nearly five times higher than younger (April) instars of *Allogamus* (0.005 ± 0.001 k/shredder/day), which showed the lowest rate. Within shredder combinations, the *Sericostoma* + *Potamophylax* pair in April showed the highest decomposition rate among mixtures (0.016 ± 0.001 k/shredder/day) (Figure 1).

Coherently, FPOM production rates varied depending on the shredder treatment: month interaction (GLM, *p* < 0.05) (Figure 1 and Table S2). Intraspecific differences in FPOM production were observed for *Potamophylax*, with younger (April) instars showing significantly higher production than older (May) instars (7.31 ± 0.88 vs. 4.39 ± 0.87 mg/shredder/day; Tukey‐adjusted, *p* = 0.028). In contrast, *Allogamus*, *Sericostoma* and all shredder combinations did not differ significantly between April and May (Table S2). Among all treatments, younger (April) instars of *Potamophylax* had the highest FPOM production overall, which was 10 times higher than that of older (May) *Allogamus* instars, which had the lowest FPOM production (0.73 ± 0.12 mg/shredder/day) (Figure 1). Among shredder combinations, older (May) instars of the *Allogamus + Sericostoma* pair had the lowest FPOM production rates (1.19 ± 0.64 mg/shredder/day), while the younger (April) instars of the *Sericostoma + Potamophylax* pair exhibited the highest rates (4.89 ± 0.47 mg/shredder/day) (Figure 1).

For FCE, there were significant differences among shredder treatments (*F*_(6,56)_ = 3.081, *p* < 0.05; Table 1). In contrast, no significant differences were observed between the instar stages across months, nor was there a significant shredder × instar month interaction. FCE varied among taxa and instars, but statistical differences were only observed between *Allogamus* and *Potamophylax*. Younger (April) instars of *Potamophylax* showed an FPOM conversion efficiency (FCE) of 59.97% ± 10.64%, while younger (April) instars of *Allogamus* had an FCE of 8.95% ± 13.60% (Figure 1 and Table S2). Among the shredder combinations, older (May) instars of *Allogamus* + *Sericostoma* had the lowest FCE (25.51% ± 15.29%), whereas the treatment with all three shredder taxa (*Allogamus*, *Potamophylax*, *Sericostoma*) in May exhibited the highest efficiency (47.64% ± 7.15%; Figure 1c).

**FIGURE 2:**
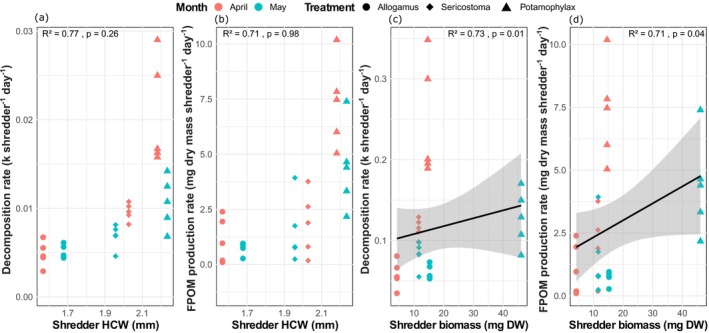
Linear relationships between decomposition rates and shredder head capsule width (a), FPOM production rates and shredder head capsule width (b), decomposition rates and shredder biomass (c), and FPOM production rates and shredder biomass (d) across different shredder taxa and larval instars sampled in April (younger instars) and May (older instars). FPOM = Fine Particulate Organic Matter, HCW = Head capsule width.

Both decomposition rates and total FPOM production increased with shredder biomass and HCW (Figure 2). *Potamophylax* taxa, with the largest HCW and highest biomass (Figure S2), had the highest decomposition and FPOM production rates. Shredder biomass had a significant effect on leaf processing, showing strong significant relationships with both decomposition and FPOM production rates (Figure 2).

**FIGURE 3:**
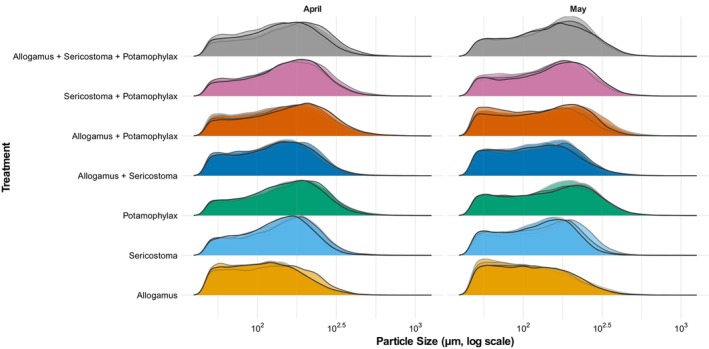
Particle size distribution of fine particulate organic matter across various treatments. The color under the graph denotes the particle density distribution under different treatment groups, while the outlines represent the replicates within each treatment.

The distribution of FPOM particle sizes differed among treatments (Table 2). With the exception of the *Allogamus* treatment, a bimodal distribution of FPOM particle sizes was observed across all treatments. Younger (April) instars of *Potamophylax* exhibited the widest particle size distribution compared to the other single treatments, with a higher proportion of larger particles (Figure 3). In contrast, the *Allogamus* treatment showed a distribution skewed toward smaller particles compared to all other treatments. The *Sericostoma* treatment also showed a distribution skewed toward larger particles but with a smaller proportion of large particles than *Potamophylax*, placing it between the *Potamophylax* and *Allogamus* single‐shredder treatments. Shredder combinations that included *Potamophylax* produced a broader FPOM particle size distribution than those without it, while the *Allogamus* + *Sericostoma* combination resembled the *Sericostoma* treatment (Figure 3).

**TABLE 2:** Summary statistics of mean FPOM particle sizes (± SD μm) in different treatments.

Treatments	Instar	
	April	May
*Allogamus*	124.74 ± 68.57^a,A^	125.66 ± 76.14^a,A^
***Sericostoma***	**152.55 ± 76.02** ^**a,ABC**^	**139.79 ± 72.13** ^**b,AB**^
*Potamophylax*	169.97 ± 93.34^a,B^	169.46 ± 99.06^a,C^
*Allogamus + Sericostoma*	144.04 ± 77.65^a,AC^	135.65 ± 74.46^a,AB^
*Allogamus + Potamophylax*	166.29 ± 99.63^a,BC^	155.25 ± 92.62^a,BC^
*Sericostoma + Potamophylax*	168.99 ± 91.63^a,B^	160.27 ± 87.03^a,BC^
*Allogamus + Sericostoma + Potamophylax*	162.76 ± 90.82^a,BC^	169.50 ± 89.84^a,C^

*Note:* Bolded treatments with lowercase letters indicate significant intraspecific differences between younger (April) and older (May) instars within each treatment. Uppercase letters indicate significant interspecific differences among shredder treatments within each month.

The FPOM particle sizes produced during decomposition differed significantly among shredder treatments (*F*_(6,56)_ = 36.65, *p* < 0.05) and between instars at different months (*F*_(1,56)_ = 5.82, *p* < 0.05) and there was no significant interaction between shredder type and instar month (*F*_(6,56)_ = 1.94, *p* = 0.091; Table 1). FPOM particle sizes were generally larger for younger (April) instars than older (May) instars in most treatments, except in *Allogamus* and the three‐taxa treatment, where the trend was reversed but not statistically significant. Only *Sericostoma* showed a statistically significant difference between months. *Potamophylax* produced the largest FPOM particles on average, particularly in the younger instars in April (169.97 ± 93.34 μm), while *Allogamus* produced the smallest mean FPOM particles (124.74 ± 68.57 μm) in younger (April) instars (Table 2). A similar trend was observed in the shredder combinations, with the younger (April) instars producing larger FPOM particles than the older (May) instars, except in the combination including all three shredder taxa. Among the shredder combinations, the treatment involving all shredder taxa combinations *Allogamus + Sericostoma + Potamophylax* in May produced the largest FPOM particles (169.50 ± 89.84 μm), while the *Allogamus* + *Sericostoma* shredder combination in May yielded the smallest mean FPOM particle sizes (135.65 ± 74.46 μm; Table 2).

**FIGURE 4:**
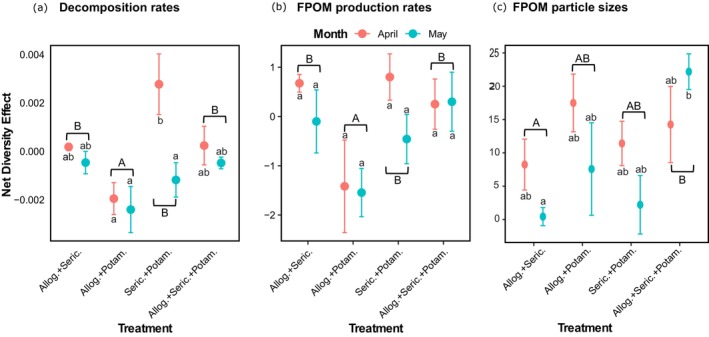
Biomass‐corrected net diversity effects on decomposition (a), FPOM production rates (b), and FPOM particle sizes (c) across treatments in the younger (April) and older (May) instar stages. Bars represent mean ± SEM (standard error of the mean). Different lowercase letters indicate significant differences among all “shredder treatment: month” interactions, while uppercase letters denote differences among shredder treatments. Allog. + Seric. = *Allogamus* + *Sericostoma*, Allog. + Potam. = *Allogamus* + *Potamophylax*, Seric. + Potam. = *Sericostoma* + *Potamophylax*, Allog. + Seric. + Potam. = *Allogamus* + *Sericostoma* + *Potamophylax*, FPOM = Fine Particulate Organic Matter.

We observed variability in net diversity effects on both decomposition and FPOM production across treatments (Figure 4). For decomposition, only the *Sericostoma + Potamophylax* treatment showed a significant difference between instars, with younger (April) instars exhibiting a positive net diversity effect and older (May) instars showing a negative one. *Allogamus + Sericostoma* and the full three‐shredder taxa mix showed no net diversity effect in either month. In contrast, the *Allogamus + Potamophylax* combination consistently showed negative effects across both months (Figure 4a).

Similarly, for FPOM production, the *Allogamus + Potamophylax* treatment showed negative net diversity effects for both younger (April) and older (May) instars. *Sericostoma + Potamophylax* showed a positive effect in the younger (April) instars but a negative one in older (May) instars. Positive effects were also observed in younger (April) instars of *Allogamus + Sericostoma* and *Sericostoma + Potamophylax*, and in both months for the three‐taxa combination (Figure 4b).

For FPOM particle sizes, net diversity effects were generally higher in younger (April) instars than in the older (May) instars, except in the three‐taxa combination (Figure 4c). Notably, all treatments showed positive net diversity effects, indicating that mixed‐shredder treatments produced larger FPOM particles than expected from the average of single‐shredder taxa treatments, and these effects were stronger overall than those observed for decomposition and FPOM production. The highest effects were recorded in the older (May) instars of the three‐taxa combination (Figure 4c).

**FIGURE 5:**
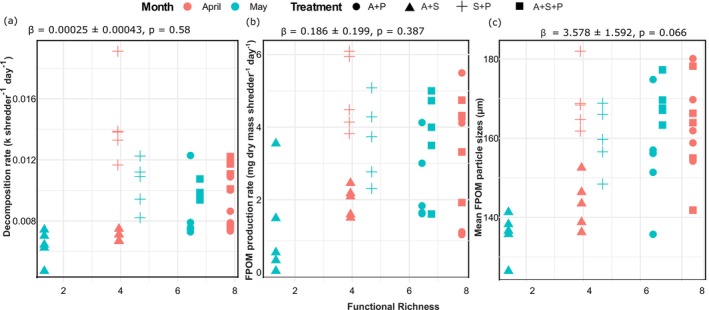
Relationship between functional richness (FRic) and ecosystem process responses: (a) decomposition rate (k, per shredder per day), (b) FPOM production rate (mg dry mass shredder^−1^ day^−1^), and (c) FPOM particle size (μm), across treatments and sampling months. FRic was calculated from biomass and head capsule width traits. Points represent treatment means, colored by month and shaped by treatment. The black dashed line represents the overall linear regression fit. FPOM = Fine Particulate Organic Matter.

The linear mixed‐effects model including Treatment × Month as a random effect showed that functional richness (FRic) was not a predictor of decomposition rate (*β* = 0.00025 ± 0.00043, *t* = 0.58, *p* = 0.58; Table 3). The random effect of Treatment × Month accounted for approximately 69.5% of the total variance (*σ* ^2^ = 6.21 × 10^−6^ vs. residual *σ* ^2^ = 2.72 × 10^−6^; Table 3 and Figure 5).

**TABLE 3:** Results of linear mixed‐effects models examining the effects of functional richness (FRic) on decomposition rate, FPOM production, and mean FPOM particle sizes.

Response variables	Fixed effect	Estimate	Std. error	df	*t*	*p*	Random effect Variance	Residual Variance	% variance (Treatment: Month)
Decomposition rate (k shredder^−1^ day^−1^)	Intercept	0.00816	0.00248	6	3.29	**0.0166**	6.21 × 10^−6^	2.72 × 10^−6^	69.50%
	FRic	0.00025	0.00043	6	0.58	0.581			
FPOM production (mg shredder^−1^ day^−1^)	Intercept	2.0288	1.1467	6	1.77	0.127	1.113	1.656	40.20%
	FRic	0.186	0.1993	6	0.93	0.387			
Mean FPOM particle sizes (μm)	Intercept	138.148	9.16	6	15.082	**< 0.001**	74.47	88.39	45.70%
	FRic	3.578	1.592	6	2.248	0.066			

*Note:* Statistically significant (*p* < 0.05) values are shown in bold. Fixed effects include FRic, and random effects include Treatment × Month. % Variance indicates the proportion of total variance explained by the random effect.

Similarly, functional richness did not predict FPOM production rates (*β* = 0.186 ± 0.199, *t* = 0.47, *p* = 0.387; Table 3). The random effect of Treatment × Month accounted for ~40% of total variance (*σ* ^2^ = 1.113 vs. residual *σ* ^2^ = 1.656; Table 3 and Figure 5).

For mean observed FPOM particle sizes (μm), functional richness showed a marginal but not significant positive effect (*β* = 3.578 ± 1.592, *t* = 2.25, *p* = 0.066; Table 3). The random effect of Treatment × Month accounted for 45.7% of the total variance (*σ* ^2^ = 74.47 vs. residual *σ* ^2^ = 88.39; Table 3 and Figure 5).

## 4 Discussion

We tested how shredder identity, ontogenetic stage, and shredder diversity influence decomposition, FPOM production, and FPOM particle size distribution. Grounded in trait‐based ecology, we hypothesized that shredder identity and body size would strongly influence processing rates, with younger larval instars and larger taxa exhibiting higher decomposition and FPOM production due to metabolic and size‐related advantages, and that greater shredder diversity would enhance processing efficiency through functional complementarity. Our results partially support these hypotheses and provide insights into how intraspecific (ontogenetic) and interspecific (taxa) trait variation shapes ecosystem functioning. Across taxa, larger taxa, such as *Potamophylax*, exhibited higher decomposition rates, FPOM production, and larger FPOM particle sizes, while smaller taxa like *Allogamus* showed lower rates and narrower size distributions, in line with our expectations regarding interspecific differences. Within taxa, however, younger and smaller instars exhibited higher decomposition rates and FPOM production rates than older, larger instars, consistent with our predictions about ontogenetic effects. Diversity effects were less consistent and varied depending on shredder combination and developmental stage, with some combinations enhancing and others reducing functional outcomes, likely due to competitive interactions or selection effects. Consistent with this, functional richness (FRic) showed no significant relationships with decomposition or FPOM production and only a marginal positive effect on FPOM particle size, offering limited support for the hypothesis that greater shredder diversity and broader trait space enhance leaf processing efficiency. Overall, our findings emphasize that shredder taxon identity and ontogenetic stage are key drivers of ecosystem processes, as these traits directly determined decomposition and FPOM production rates. In contrast, diversity and functional richness influenced ecosystem processes only under certain treatment and taxa combinations, indicating that their effects are context‐dependent and mediated by taxa composition and ontogenetic structure.

### 4.1 Decomposition Rates and FPOM Production

We interpret the observed differences in decomposition rates and FPOM production as reflecting size‐mediated taxon‐specific feeding behavior, physiology, and efficiency in processing leaf litter (Patrick 2013; Tonin et al. 2018; Balik et al. 2022). The interspecific differences align with previous findings that have proposed not only a link of mouthpart and digestive tract anatomy but also digestive capacity to variations in organic matter processing (Labandeira 1997; Weiss 2006; Friedrich et al. 2015; Zittra et al. 2022). Ontogenetic stage further modulated these taxon‐specific traits, with younger instars showing relatively higher decomposition and FPOM production rates. We attribute these observations to the higher metabolic rates and more opportunistic feeding behavior of the smaller early developmental stages (Brown et al. 2004; Céréghino 2006; Katano et al. 2017): younger larvae must maximize consumption to meet energy demands, which—in shredders—accelerates organic matter breakdown and particle generation (Anderson 1979; Perry et al. 1987).

Conversely, the reduced FPOM production of older instars likely reflects a shift in feeding strategy, as these stages need to optimize their essential nutrient: biomass ratio (Slansky and Scriber 1985; Wagner 1990). Indeed, such presumed changes in feeding ecology plausibly explain the observed shifts in decomposition efficiency (Bohle 1983; Werner and Gilliam 1984; Hildrew et al. 2007). Moreover, the interactions of the shredder taxa in specific ontogenetic stages returned variable diversity effects on decomposition and FPOM production rates: Positive net diversity effects on FPOM production occurred in combinations involving *Allogamus + Sericostoma, Sericostoma + Potamophylax*, and the full shredder taxa mix, especially with younger instars. These outcomes suggest complementary feeding strategies enhancing resource use and decomposition. In contrast, the *Allogamus + Potamophylax* pair exhibited negative net diversity effects in decomposition and FPOM production rates. This pattern may indicate competition or interspecific interference between these two closely related species, which could have resulted in negative diversity effects (Dudley et al. 1990; Amarasekare 2002), despite both belonging to the same family.

Net diversity effects were generally higher in younger instars compared to older instars, possibly due to, again, an ontogenetic‐related shift in feeding ecology. Interestingly, net diversity effects were also not universally positive; we also observed negative effects, likely reflecting strong selection in combinations such as *Sericostoma + Potamophylax* where younger (April) instars showed a positive net diversity effect, whereas older (May) instars showed a negative effect. These patterns indicate that selection effects may become stronger with larval age across shredder combinations. This is supported by observations of functional richness, where in most cases, younger instars had higher functional richness than their older conspecifics. Ontogenetic shifts toward selective feeding and size‐based competitive dominance likely reinforce these patterns, where larger individuals reduce complementarity by outcompeting smaller or younger specimens (Loreau and Hector 2001; Boyero et al. 2007; Dangles et al. 2011). In other cases, such as the *Allogamus + Potamophylax* combination, negative effects were observed across both months for decomposition and FPOM production. Here, a dominant taxon (*Potamophylax*) largely contributed to decomposition and FPOM production, which may have limited the relative contribution of the less efficient partner. Such dominance can mask potential complementarity, especially when species share similar niches or compete for limited resources (Werner and Gilliam 1984; Wellnitz and Poff 2001; Pastore et al. 2021). The complexity of diversity effects observed here challenges the assumption that increased diversity always enhances ecosystem function, echoing previous research on competition and redundancy in stream communities (Wellnitz and Poff 2001; Pacini et al. 2024). Furthermore, functional richness metrics revealed no significant relationships with decomposition rate or FPOM production. Overall, these patterns appear to be influenced more by taxonomic identity than by the overall functional richness of the assemblages, suggesting that taxonomic composition has a stronger effect on decomposition and FPOM production. At the same time, intraspecific differences among instars and the associated variation in functional traits further shape net diversity effects, highlighting the combined influence of inter‐ and intraspecific variation on ecosystem functioning.

### 4.2 FPOM Particle Sizes

The size distribution of FPOM particles produced by shredders was strongly shaped by taxon identity and largely reflected differences in body size. Greater variability observed in the larger taxa likely stems from differences in mouthpart morphology and feeding mechanics that produce a wider particle size spectrum (Patrick 2013; Tonin et al. 2018). Supporting this, Krings et al. (2021) found a positive correlation between body mass and radular force in gastropods, linking larger body size to greater feeding capacity and potential particle fragmentation. Reduced particle size variability, in contrast, suggests a finer, more targeted processing strategy employed by smaller individuals (Tonin et al. 2018). Across most treatments, a bimodal FPOM particle size distribution was observed, likely reflecting two major FPOM sources: larger mechanically shredded leaf fragments and smaller fecal particles. Notably, our particle size data also revealed strong selection exerted by the most efficient shredder taxon: Combinations containing *Potamophylax* exhibited FPOM distributions similar to *Potamophylax* alone—the taxon that had the highest decomposition and FPOM production rates—suggesting efficient resource use and complementarity via resource partitioning (Vos et al. 2011). A similar trend was observed in *Sericostoma* + *Allogamus* combinations, where distributions resembled those of single *Sericostoma* treatments, indicating non‐additive effects in mixed assemblages. These outcomes highlight how high‐performing taxa can dominate functional outputs of diverse communities.

Clearly, body size is a critical functional trait and mediates effects of shredder diversity on leaf litter decomposition in streams (Reiss et al. 2011; Patrick 2013; Tonin et al. 2018). At the same time, the direction and magnitude of size‐controlled differences among taxa depend strongly on ontogenetic stage. It is plausible that higher net diversity effects observed in younger instars relate to more complementary functional roles of these stages—likely due to broader feeding niches, lower specialization, and more flexible resource use. For example, *Potamophylax* larvae construct leaf cases when young but shift to stone cases as they mature (Otto and Svensson 1980) and thus change resource use, which may help overcome niche overlap of younger and older instars—but also changes FPOM production dynamics.

Consistent with this, net diversity effects were even more pronounced for FPOM particle sizes, where older instars in the three‐taxa mixture showed the strongest net diversity effects despite their lower functional richness compared to their younger conspecifics. This pattern again points toward shredder interaction‐driven processes, such as increased feeding selectivity and competitive dominance in larger individuals, rather than trait richness alone determining particle‐size outcomes. Such ontogenetic contrasts reflect broader patterns in natural communities, where size hierarchies can suppress the functional contribution of smaller taxa, reinforcing redundancy and limiting positive biodiversity effects (Creed et al. 2009; Balik et al. 2022). These findings raise an important question: to what extent can different species compensate for one another's functional roles in stream ecosystems and at which ontogenetic stage?

While multiple shredder species contribute to decomposition and FPOM production at different ontogenetic stages, their roles are obviously not interchangeable. Clear differences in decomposition rates, FPOM production, and particle size distributions—especially the high contributions from *Potamophylax* in the younger (April) instars—suggest that full functional replacement is unlikely. Additionally, the efficiency of organic matter decomposition is shaped by environmental conditions and resource availability (Ferreira et al. 2020; Mas‐Marti et al. 2015).

### 4.3 Implications for Ecosystem Functioning

Higher temperatures, for instance, are known to accelerate metabolic rates and larval development in aquatic insects, leading to earlier emergence or pupation at smaller body sizes (Hogg and Williams 1996; McCauley et al. 2015). This may shorten, but intensify active decomposition periods and possibly reduce FPOM production via a shortened overall period of activity. Altered phenology could also create mismatches between shredder activity and CPOM input, decoupling organic matter processing from seasonal litterfall and disrupting stream metabolism (Boyero et al. 2012; Altermatt 2013). At the level of FPOM particle sizes, shredder diversity—encompassing taxonomic richness, functional traits, and ontogenetic development (Loreau and Hector 2001; Boyero et al. 2007)—is particularly relevant. The gradient in particle size produced by different taxa affects the quality and availability of detrital resources for downstream consumers and influences energy flow and nutrient cycling in stream ecosystems (Bundschuh and McKie 2016). For example, dominance by large taxa producing larger FPOM particles may limit downstream transport and restrict resources for filter‐feeders and collector‐gatherers adapted to finer particles (Wallace and Grubaugh 1996; de Paula et al. 2016). Conversely, dominance by smaller taxa may support downstream food webs through increased transport of finer FPOM particles. Consequently, the loss of high‐function taxa or taxa of a particular size may lead to significant declines in ecosystem functioning, with redundancy acting as a buffer in some cases—where species or larval stages perform similar roles—but as a bottleneck in others, where unique functions provided by key species cannot be replaced.

Ontogenetic differences may also play a buffering role: older taxa with larger mouthparts can consume more recalcitrant leaf litter, while smaller instars process labile material earlier, potentially extending decomposition over time. The observation that FPOM particle size additionally depends on initial CPOM quality adds another level of complexity to this supposedly simple ecosystem function (Yegon et al. unpublished data). In this light, it appears possible that benthic invertebrate shredder diversity may counteract effects of riparian biodiversity loss or shifts in riparian vegetation that reduce CPOM quality. Moreover, the opposite—diversity of riparian vegetation buffering against benthic invertebrate shredder loss—also appears as a distinct possibility. In total, CPOM quality and quantity, shredder diversity, shredder community structure and ontogeny must therefore be understood as key factors influencing both the intensity and quality of organic matter processing in stream ecosystems (Werner and Gilliam 1984).

In light of these considerations, it is important to acknowledge the limitations of our work. For instance, developmental progression in some taxa, particularly *Sericostoma*, coincided with the onset of pupation, which prevented consistent sampling of the largest larvae in late spring. As a result, the May samples also included relatively smaller individuals, contributing to greater variability in HCW and biomass. While this does not affect the overall patterns observed, it may partly explain unexpected size differences across sampling periods and highlights natural variability in growth within instars and the challenges of sampling across ontogenetic stages. Some other caveats remain, particularly regarding the taxonomic and experimental breadth of our design. Although we included both distantly related taxa (different families: Sericostomatidae vs. Limnephilidae) and more closely related taxa (*Allogamus*/*Potamophylax*, both Limnephilidae), the overall taxonomic richness was restricted to three. This limited diversity constrains the generality of our conclusions and may reduce the range of functional differences detectable across taxa. In addition, the microcosm approach, while allowing strong experimental control, necessarily simplifies ecological complexity compared to natural stream communities, potentially influencing species interactions and ecosystem processes. Also, while our study focused on selected shredder taxa and their larval instars, natural communities display far greater ontogenetic and taxonomic diversity, corresponding to a wider spectrum of functional traits that shape ecosystem functioning. Despite these constraints, our findings highlight that shredder identity strongly influenced litter decomposition, FPOM production, and particle size diversity, suggesting that taxonomic identity can be an important driver of biodiversity–ecosystem functioning (BEF) relationships in detritus‐based systems. Future work, covering a broader array of taxa and more complex community structures against a more comprehensive resource background, would help assess the generality of these patterns under natural conditions.

To conclude, our results demonstrate that shredder identity, ontogenetic stage, and diversity that represent functional trait gradients shape organic matter processing in a complex, nonlinear way. This complexity challenges the assumption of functional equivalence among related taxa (cf. Heino et al. 2007; Heino 2010). Traits such as body size, developmental stage, and feeding behavior not only determine how efficiently shredders break down litter but also mediate their interactions, influencing decomposition outcomes. Biodiversity effects on this function are therefore shaped less by species richness alone than by trait complementarity, resource use, and competitive dynamics. Ontogenetic shifts further modulate these interactions by altering feeding behavior and efficiency, creating nonlinear BEFs driven primarily by species compatibility and functional traits. Future research should test these patterns in natural stream communities, explore the variation in the underlying functional traits and examine whether functional redundancy may buffer the effects of biodiversity loss.

## Author Contributions

**Mourine J. Yegon:** conceptualization (equal), data curation (lead), formal analysis (lead), investigation (lead), methodology (lead), resources (equal), software (equal), validation (lead), visualization (lead), writing – original draft (lead), writing – review and editing (lead). **Pratiksha Acharya:** conceptualization (equal), data curation (equal), formal analysis (supporting), investigation (equal), methodology (equal), resources (equal), software (supporting), validation (supporting), visualization (supporting), writing – original draft (supporting), writing – review and editing (equal). **Katrin Attermeyer:** conceptualization (equal), data curation (supporting), formal analysis (supporting), funding acquisition (lead), investigation (supporting), methodology (equal), project administration (equal), resources (supporting), software (supporting), supervision (equal), validation (equal), visualization (equal), writing – original draft (supporting), writing – review and editing (equal). **Wolfram Graf:** conceptualization (equal), data curation (supporting), formal analysis (supporting), funding acquisition (supporting), investigation (supporting), methodology (equal), resources (equal), software (equal), supervision (lead), validation (supporting), visualization (supporting), writing – original draft (supporting), writing – review and editing (equal). **Simon Vitecek:** conceptualization (equal), data curation (supporting), formal analysis (equal), funding acquisition (lead), investigation (equal), methodology (equal), project administration (lead), resources (equal), software (supporting), supervision (lead), validation (equal), visualization (equal), writing – original draft (equal), writing – review and editing (equal).

## Funding

This research was supported by the State of Lower Austria through WasserCluster Lunz Biological station. Open access funding provided by the University of Natural Resources and Life Sciences, Vienna (BOKU).

## Conflicts of Interest

The authors declare no conflicts of interest.

## Supporting information


**Data S1:** ece372907‐sup‐0001‐Supinfo01.docx.

## Data Availability

Data for this study are provided and are accessible under this private link on Figshare repository (https://figshare.com/s/ef56ace847bfa6e447aa) for the peer review. Should the manuscript be accepted, the link to the data will be made public.
