# Supplementary material for: Divergent Functional Roles of Shredder Size: Interspecific Versus Intraspecific Effects on Aquatic Leaf Litter Decomposition
Source: Ecol Evol. 2026 Jan 12;16(1):e72907. doi: 10.1002/ece3.72907 (PMC12795619; doi:10.1002/ece3.72907)
Supplement: Supplementary file 1 — Data S1: ece372907‐sup‐0001‐Supinfo01.docx. [file ECE3-16-e72907-s001.docx]

**Supplementary material**

**Divergent functional roles of shredder size: interspecific versus intraspecific effects on aquatic leaf litter decomposition**

Mourine J. Yegon^1, 2*^, Pratiksha Acharya^1, 3^, Katrin Attermeyer^1, 3^, Wolfram Graf^2^ & Simon Vitecek^1,2,4*^

^1^Wassercluster Lunz - Biological Station, Dr. Carl Kupelwieser-Prom. 5, 3293, Lunz am See, Austria

^2^Institute of Hydrobiology and Aquatic Ecosystem Management, University of Natural Resources and Life Sciences, Gregor-Mendel-Straße 33/DG, 1180, Vienna, Austria

^3^Department of Functional and Evolutionary Ecology, University of Vienna, Djerassiplatz 1, 1030 Vienna, Austria

^4^Department of Ecology, University of Innsbruck, Innsbruck, Austria

*Corresponding author email: [mourine.yegon@wcl.ac.at](mailto:mourine.yegon@wcl.ac.at); simon.vitecek@boku.ac.at

**Supplementary Table 1:** *Experimental replication design*

| **Scale of inference** | **Scale at which factor of interest is applied** | **Number of replicates at the appropriate scale** |
| --- | --- | --- |
| Microcosms | macroinvertebrate shredders | 70 (7 treatments × 2 months × 5 replicates) |
| Shredder density | microcosms | 12 per microcosm (12 × 70 = 840) |
| Shredder identity | microcosms | 5 replicates per identity × 2 months = 10 replicates per identity |
| Shredder diversity level | microcosms | 5 replicates per level × 2 months = 10 per diversity level (single, pairwise, triple) |
| Ontogenetic stage (month) | month | 2 (April, May) |

**Supplementary Table 2:** *Pairwise comparisons of decomposition rates (K/shredder/day), FPOM production rates (mg dry mass/shredder/day), and FPOM conversion efficiency (%) between April (younger instars) and May (older instars) within each shredder treatment. Comparisons are based on estimated marginal means (~ Month | Treatment) from GLMs with Tukey adjustment. Columns show the mean ± SE for each month, the contrast (April − May), standard error (SE), degrees of freedom (df), t-ratio, and p-value. P-values in bold indicate significant differences (p < 0.05) between April and May within the same treatment.*

| **Treatment** | **April_Mean** | **May_Mean** | **Estimate** | **SE** | **df** | **t-statistic** | **p-value** |
| --- | --- | --- | --- | --- | --- | --- | --- |
| **Decomposition rates** |  |  |  |  |  |  |  |
| *Allogamus* | 0.0048 ± 0.001 | 0.0051 ± 0.0004 | -0.0791 | 0.125 | 56 | -0.635 | 0.5283 |
| *Sericostoma* | 0.0096 ± 0.0001 | 0.0068 ± 0.001 | 0.354 | 0.125 | 56 | 2.839 | **0.0063** |
| *Potamophylax* | 0.0206 ± 0.003 | 0.0106 ± 0.001 | 0.6577 | 0.125 | 56 | 5.276 | **<0.0001** |
| *Allogamus+Sericostoma* | 0.007 ± 0.0001 | 0.0064 ± 0.0001 | 0.1071 | 0.125 | 56 | 0.859 | 0.3941 |
| *Allogamus+Potamophylax* | 0.009 ± 0.001 | 0.0085 ± 0.001 | 0.0621 | 0.125 | 56 | 0.498 | 0.6204 |
| *Sericostoma+Potamophylax* | 0.0144 ± 0.001 | 0.0104 ± 0.001 | 0.3177 | 0.125 | 56 | 2.549 | **0.0136** |
| *Allogamus+Sericostoma+Potamophylax* | 0.0106 ± 0.001 | 0.0098 ± 0.0001 | 0.0588 | 0.125 | 56 | 0.471 | 0.6393 |
|  |  |  |  |  |  |  |  |
| **FPOM production rates** |  |  |  |  |  |  |  |
| *Allogamus* | 1.1164 ± 0.46 | 0.7306 ± 0.12 | 0.09461 | 0.279 | 56 | 0.339 | 0.7358 |
| *Sericostoma* | 1.8512±0.64 | 1.4978±0.66 | 0.13704 | 0.279 | 56 | 0.491 | 0.6252 |
| *Potamophylax* | 7.3065±0.88 | 4.3857±0.87 | 0.63026 | 0.279 | 56 | 2.259 | **0.0278** |
| *Allogamus+Sericostoma* | 1.9657±0.18 | 1.1913±0.64 | 0.47297 | 0.279 | 56 | 1.695 | 0.0956 |
| *Allogamus+Potamophylax* | 2.5715±0.94 | 2.4437±0.49 | -0.035 | 0.279 | 56 | -0.126 | 0.9005 |
| *Sericostoma+Potamophylax* | 4.8948±0.47 | 3.6358±0.5 | 0.3142 | 0.279 | 56 | 1.126 | 0.2649 |
| *Allogamus+Sericostoma+Potamophylax* | 3.7165±0.51 | 3.7651±0.6 | -0.0028 | 0.279 | 56 | -0.01 | 0.9922 |
|  |  |  |  |  |  |  |  |
| **FPOM conversion efficiency** |  |  |  |  |  |  |  |
| *Allogamus* | 8.9475±13.597 | 16.9311±3.301 | 12.02 | 14.4 | 56 | 0.837 | 0.4062 |
| *Sericostoma* | 24.4058±8.232 | 30.7287±16.667 | -6.32 | 14.4 | 56 | -0.44 | 0.6614 |
| *Potamophylax* | 59.9684±10.638 | 54.8001±10.593 | 5.17 | 14.4 | 56 | 0.36 | 0.7202 |
| *Allogamus+Sericostoma* | 32.9765±2.961 | 25.5128±15.295 | 7.46 | 14.4 | 56 | 0.52 | 0.6052 |
| *Allogamus+Potamophylax* | 35.4973±12.467 | 36.9948±8.665 | -1.5 | 14.4 | 56 | -0.104 | 0.9173 |
| *Sericostoma+Potamophylax* | 47.4902±3.587 | 46.2893±8.672 | 1.2 | 14.4 | 56 | 0.084 | 0.9336 |
| *Allogamus+Sericostoma+Potamophylax* | 46.5364±7.991 | 47.6464±7.151 | -1.11 | 14.4 | 56 | -0.077 | 0.9386 |

**Supplementary Figure 1:** *Experimental setup. The figure includes (A) a schematic drawing of the microcosm design, (B) a photograph showing the microcosms in the climate-controlled chamber, and (C) the insects used in each microcosm, including their stocked densities and their mortalities in the months of April and May. Different panels in C indicate the various shredder taxa and combinations used in the treatments.*

**
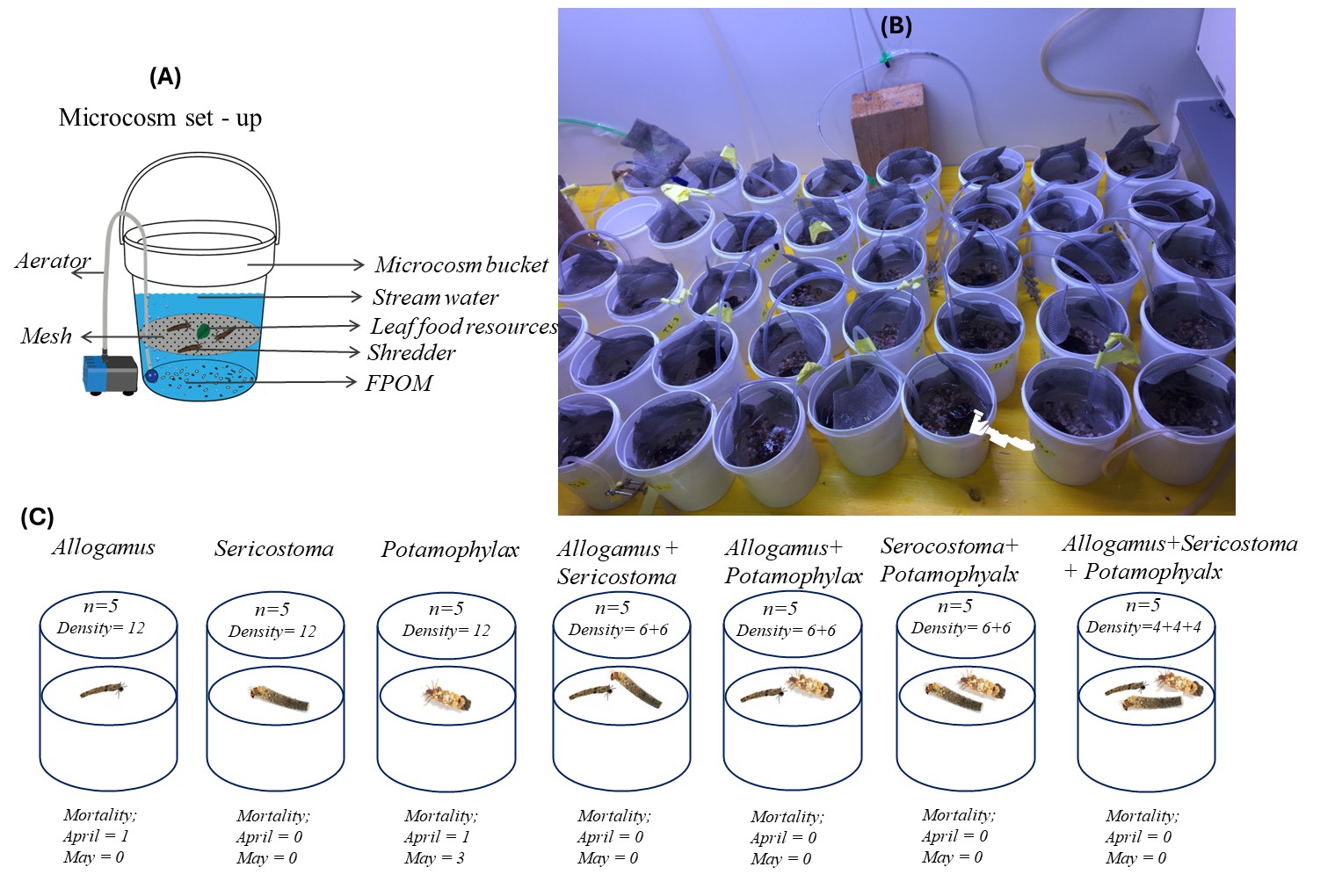
**

**Supplementary Figure 2:** *Size differences of shredder instars across months, measured by head capsule width (A) and dry mass (B). Lowercase letters indicate significant differences between April and May instars within each treatment, while uppercase letters denote differences among shredder treatments. n = 10 per shredder per treatment per month. HCW = head capsule width, DW= dry weight.*

**
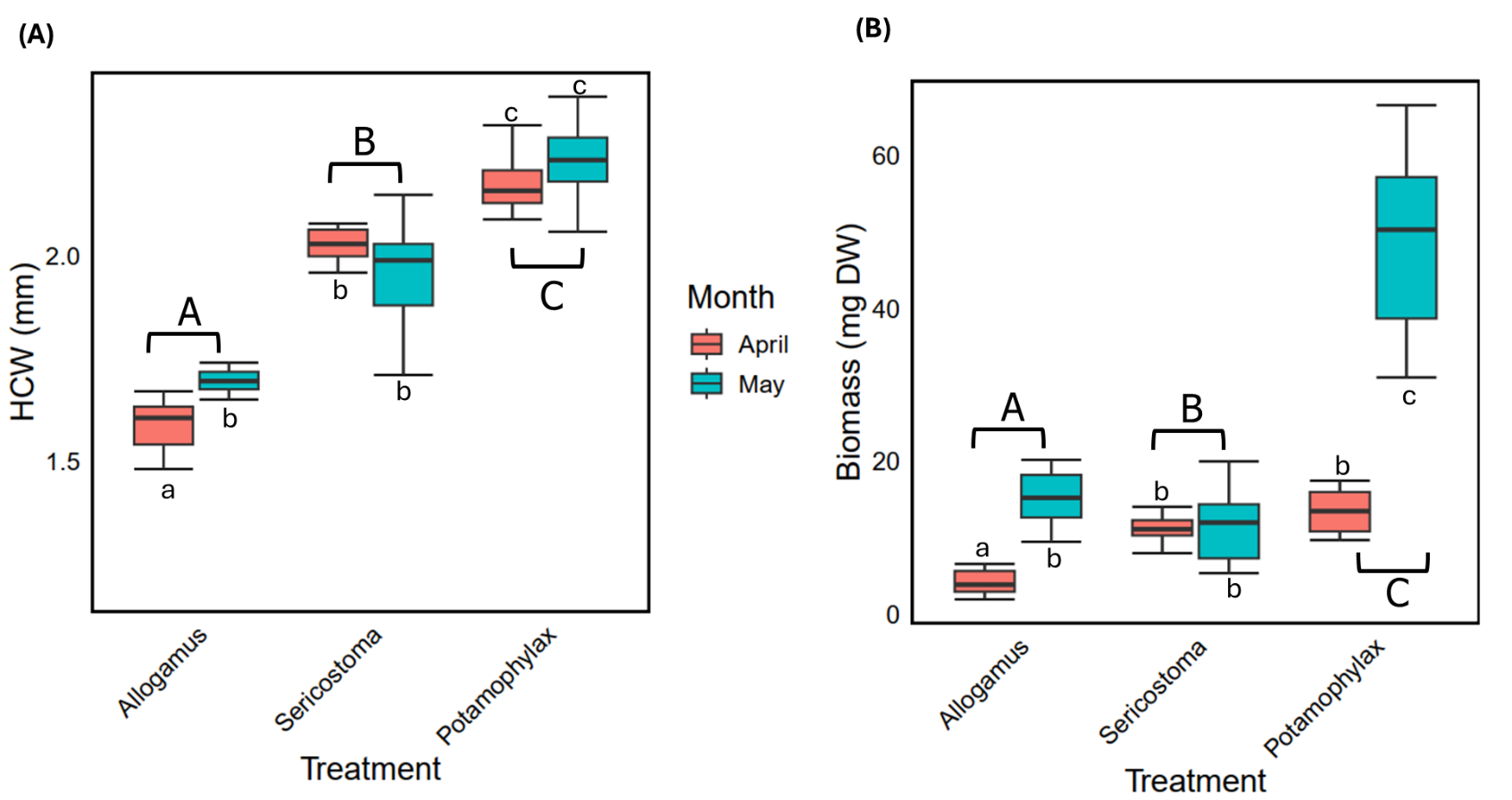
**
